# Histopathological Characterization and Whole Exome Sequencing of Ectopic Thyroid: Fetal Architecture in a Functional Ectopic Gland from Adult Patient

**DOI:** 10.1155/2018/4682876

**Published:** 2018-02-08

**Authors:** Rosalinda Yasato Camargo, Cristina Takami Kanamura, Celso Ubirajara Friguglietti, Célia Regina Nogueira, Sonia Iorcansky, Alfio José Tincani, Ana Karina Bezerra, Ester Brust, Fernanda Christtanini Koyama, Anamaria Aranha Camargo, Fernanda Orpinelli R. Rego, Pedro Alexandre Favoretto Galante, Geraldo Medeiros-Neto, Ileana Gabriela Sanchez Rubio

**Affiliations:** ^1^Thyroid Unit, Cellular and Molecular Endocrine Laboratory, LIM-25, Faculdade de Medicina da Universidade de São Paulo (FMUSP), Avenida Doutor Arnaldo 455, Cerqueira César, 01246-904 São Paulo, SP, Brazil; ^2^Adolfo Lutz Institute, São Paulo Public Health Service, Av. Dr. Arnaldo 355, Cerqueira César, 01246-000 São Paulo, SP, Brazil; ^3^Head and Neck Surgery of Santa Catarina Hospital, Av. Paulista 200, Bela Vista, 01310-000 São Paulo, SP, Brazil; ^4^Department of Internal Medicine, Botucatu School of Medicine, UNESP, Av. Prof. Montenegro, s/n Distrito de Rubião Junior, 18618-687 Botucatu, SP, Brazil; ^5^Servicio de Endocrinología, Hospital de Pediatría Dr. Juan Garrahan, Combate de los Pozos 1881, C1245AAM Buenos Aires, Argentina; ^6^Departamento de Cirurgia na Disciplina de Cirurgia de Cabeça e Pescoço da Faculdade de Ciências Médicas da UNICAMP, R. Tessália Vieira de Camargo 126, 13083-887 Campinas, SP, Brazil; ^7^Medicine School, Universidade de Fortaleza (Unifor), Av. Washington Soares 1321, Edson Queiroz, 60811-905 Fortaleza, CE, Brazil; ^8^Postgraduate Program in Biotechnology, Universidade Federal de São Paulo (UNIFESP), Pedro de Toledo 669, 040399-032 São Paulo, SP, Brazil; ^9^Thyroid Molecular Sciences Laboratory, Universidade Federal de São Paulo, Departamento de Ciências Biológicas, Postgraduation Programs in Biotechnology and Structural and Functional Biology, UNIFESP, Pedro de Toledo 669, 040399-032 São Paulo, SP, Brazil; ^10^Molecular Oncology Center, Hospital Sírio-Libanés, Rua Prof. Daher Cutait 69, 01308-060 São Paulo, SP, Brazil

## Abstract

Ectopic thyroid results from a migration defect of the developing gland during embryogenesis causing congenital hypothyroidism. But it has also been detected in asymptomatic individuals. This study aimed to investigate the histopathological, functional, and genetic features of human ectopic thyroids. Six samples were histologically examined, and the expression of the specific thyroid proteins was assessed by immunohistochemistry. Two samples were submitted to whole exome sequencing. An oropharynx sample showed immature fetal architecture tissue with clusters or cords of oval thyrocytes and small follicles; one sample exhibited a normal thyroid pattern while four showed colloid goiter. All ectopic thyroids expressed the specific thyroid genes and T4 at similar locations to those observed in normal thyroid. No somatic mutations associated with ectopic thyroid were found. This is the first immature thyroid fetal tissue observed in an ectopic thyroid due to the arrest of structural differentiation early in the colloid stage of development that proved able to synthesize thyroid hormone but not to respond to TSH. Despite the ability of all ectopic thyroids to synthetize specific thyroid proteins and T4, at some point in life, it may be insufficient to support body growth leading to hypothyroidism, as observed in some of the patients.

## 1. Introduction

The thyroid gland is a bilobar gland composed of two endocrine cells, namely, the thyroid follicular cells (TFCs) that produce the thyroid hormones T3 and T4 and the parafollicular cells that secrete calcitonin. TFCs are derived from the thyroid primordium, or thyroid anlage, that originates from a thickening of the endodermal epithelium in the foregut at the base of the prospective tongue. This structure develops the thyroid diverticulum that proliferates, invades the surrounding mesenchyme, and migrates through the anterior midline of the neck. During migration, the primitive thyroid acquires a bilobed structure while the thyroglossal duct, the initial connection to the primitive pharyngeal floor, loses its lumen and turns into disconnected fragments. When the developing thyroid reaches its final position at the base of the neck in front of the trachea, the fusion of the ultimobranchial bodies takes place to give rise to C cells. Finally, folliculogenesis occurs concomitantly with differentiation of thyroid progenitor cells into functional thyroid follicular cells, expressing specific thyroid genes such as TSH receptor (*TSHR*), Na/I symporter (*NIS*), and thyroglobulin (*TG*) [1–3].

In mouse models, normal embryogenesis of the thyroid was shown to be controlled by a well-integrated regulatory network of transcription factors [4, 5]. During the formation of the thyroid anlage (specification phase), the expression of thyroid transcription factors TITF1, PAX8, FOXE1, and HEX1 can be observed. FOXE1 is believed to be required for migration of the mouse thyroid primordium after detachment of the bud [6], and survival of the thyroid primordium depends on the expression of FOXE1 and PAX8 [4].

Ectopic thyroid is a rare malformation that results from a migration defect of the developing gland during embryogenesis [1]. The glands can be found at any point along the path of migration, from the foramen cecum to the mediastinum, and also in distal subdiaphragmatic areas. However, the most frequent site is at the base of the tongue or lingual thyroid [7]. Ectopia is the most common form of thyroid dysgenesis (TD) and congenital hypothyroidism [8, 9] but has also been detected in asymptomatic individuals or as a cause of hypothyroidism, dysphonia, dysphagia, cough, snoring, or sleep apnea and coexisting with a normally located thyroid [10–12] [13, 14].

The pathogenesis of ectopic thyroid, and the fact that some ectopic patients remain euthyroid throughout life, has not yet been elucidated. Few studies have characterized the expression of important thyroid proteins [10, 14]. In addition, mutations in genes *FOXE1*, *PAX8*, *TSHR*, *NKX2.1*, or *NKX2.5* associated with TD were identified in only 2-3% of TD cases [15]. Recently, with whole exome sequencing (WES), a powerful tool for investigating genetic causes of human diseases, novel genes were identified in TD cases [16, 17].

Thus, the aim of the present study was to investigate the histopathological and functional characteristics of human ectopic thyroid tissues through the expression of T4, specific thyroid proteins TG, NIS, TSHR, and thyroid transcription factors NKX2.1 and PAX8 by immunohistochemistry and to identify potential pathogenic mutations with expanded whole exome sequencing.

## 2. Material and Methods

### 2.1. Samples

Paraffin blocks of four human ectopic thyroid samples were retrospectively collected from patients who underwent thyroid resection, and two flash-frozen tissue samples removed due to symptoms (sample 4) or suspicious cytology for papillary thyroid lesion (patient 2) were also included in the study. The control sample was taken from archived paraffin blocks of the normal thyroid. The study protocol was approved by the local Research Ethics Committees (HC 893/01 and CEP 1078/11).

### 2.2. Patients

All patients were born before the establishment of the mandatory Neonatal Screening Program in Brazil, except for patient 5. Clinicopathologic information on the ectopic patients was obtained from medical records. *Patient 1* is a 34-year-old female with an oropharyngeal ectopic thyroid mass detected at 7 years of age. The patient was never treated with levothyroxine (LT4) and had two cesarean deliveries. Preoperative thyroid hormonal status was TSH: 11.19 mIU/L (ref. 0.5–4.2 mIU/L), and total T4: 2.48 *μ*g/dL (ref. 5.3–12.6 *μ*g/dL) (to convert to nmol/L, multiply by 12.87). *Patient 2* is a 46-year-old female with ectopic thyroid located at the hyoid bone. The patient had been treated with LT4 (100 *μ*g/day) for hypothyroidism for the last 30 years. Goiter was diagnosed 4 years before surgery. *Patient 3* is a 7-year-old euthyroid girl. Lingual thyroid was detected at 5 years of age when investigating swallowing and sleeping difficulties. The patient was never treated with LT4. *Patient 4* is a 38-year-old male with lingual thyroid causing excessive snoring. The patient was never treated with LT4, and preoperative thyroid hormonal status was FT4: 0.6 ng/dL (ref. 0.6–1.54 ng/dL) (to convert to pmol/L, multiply by 12.87), TSH: 46.7 mIU/L (ref. 0.5–4.2 mIU/L), and negative anti-TPO and anti-Tg. *Patient 5* is a one-month-old girl with midline defect and lingual thyroid, diagnosed after surgery for suspected lingual tumor. Neonatal test after surgery indicated TSH > 200 mIU/L. *Patient 6* is a 13-year-old girl with a neck nodule noted at birth that grew during childhood development. At 11 years of age, lingual thyroid was diagnosed and hypothyroidism confirmed with serum TSH: 11.42 mIU/L, FT4: 0.73 ng/dL (ref. 0.897–1.794 ng/dL), and negative anti-TPO. Levothyroxine was prescribed, but the patient showed poor compliance.

### 2.3. Immunohistochemistry

Three *μ*m thick sections of formalin-fixed and paraffin-embedded tissue samples from the six patients (samples 1–6) were first stained with hematoxylin/eosin for histological observation and then submitted to immunohistochemical procedures. Antigen retrieval was performed in 10 mM citrate buffer/pH 6.0 in a pressure cooker for three minutes, and endogenous peroxidase was inactivated in 6% hydrogen peroxide solution in an incubation step. The antibodies used in this study were monoclonal anti-thyroid peroxidase (TPO) (MoAb47, 1 : 500, DakoCytomation, Glostrup, Denmark), monoclonal anti-sodium-iodide symporter (NIS) (FP5A, 1 : 200, Mayo Clinic, USA), monoclonal anti-TTF1 (NKX2.1) (8G7G3/1, 1 : 500, Cell Marque, Rocklin, USA), monoclonal anti-thyroid-stimulating hormone receptor (TSHR) (4C1/E1/G8, 1 : 200, NeoMarkers, Fremont, USA), polyclonal anti-PAX8 (rabbit, 1 : 500, Cell Marque), polyclonal anti-thyroglobulin (rabbit, 1 : 400.000; DakoCytomation), and polyclonal anti-thyroxine (T4) (rabbit, 1 : 1.000; Cloud-Clone Corp., Houston, USA). Tissue sections were incubated overnight with the primary antibodies, and amplifications were obtained by peroxidase-conjugated polymer (Ultravision TL015-HDS, Thermo Fisher Scientific, Fremont, USA) and then revealed by diaminobenzidine/hydrogen peroxide substrate chromogen. Images were acquired using a Leica TCS SP8 microscope. The samples were examined by two of the authors.

### 2.4. Whole Exome Sequencing and Analysis

Total DNA from ectopic tissue from patients 2 and 4 was used to prepare the DNA library with Agilent SureSelectXT reagent kit (Agilent Technologies®, Santa Clara, USA), and the whole exome and UTR regions were sequenced in Illumina NextSeq 500 platform (Illumina® Inc., San Diego, USA) with a calculated coverage average of 140x per sample. Sequenced reads were aligned to a reference genome (GRCh37/hg19) using Burrows-Wheeler Alignment (BWA), and calling was performed with Genome Analysis Toolkit (GATK). Single nucleic variants and small insertions-deletions were annotated with ANNOVAR [18]. Data were filtered with 12 sample controls of Brazilian familial thyroid cancer tissues, variants with low-quality score, and common variants (MAF > 1%), and neutral variants were eliminated. Predictions of deleterious effect were evaluated using online bioinformatics tools: SIFT, PolyPhen-2, mutationassessor, fathmm, Condel, MutationTaster, and PROVEAN [19–21].

## 3. Results

### 3.1. Histopathology of Ectopic Thyroids

**Figure 1:**
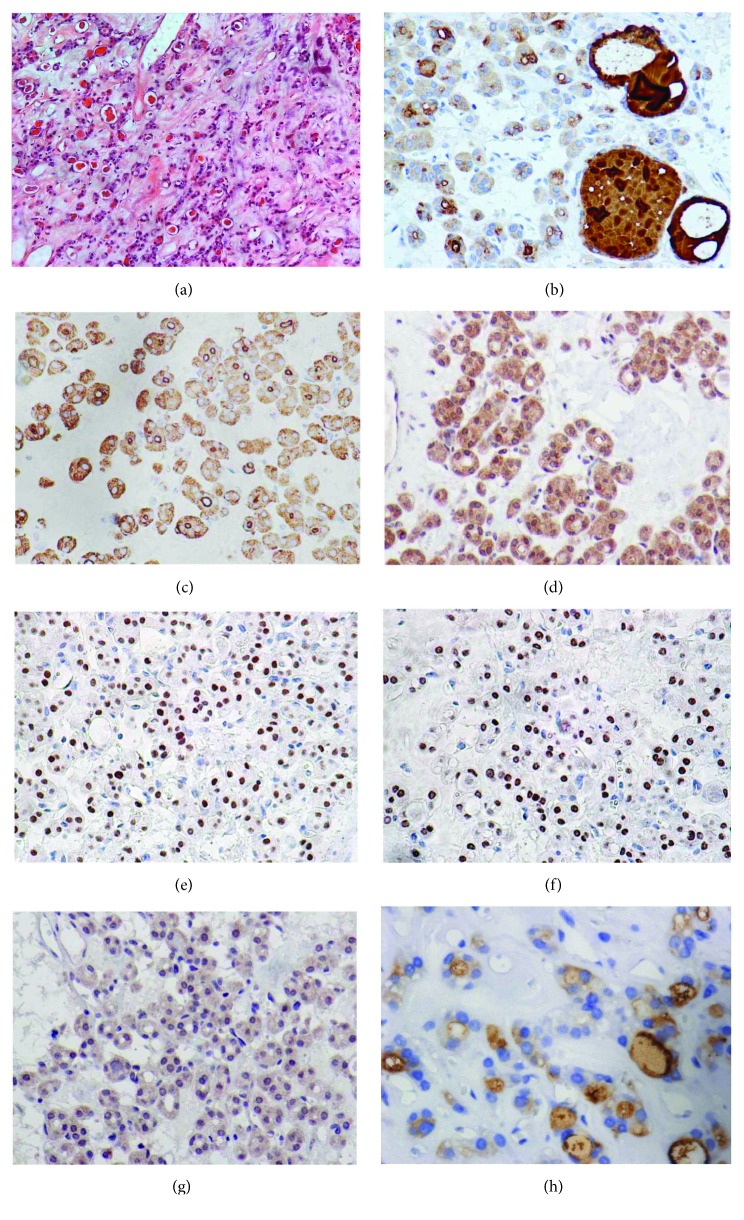
Hematoxylin/eosin staining (a) (10x) and immunohistochemistry expression of specific thyroid genes in ectopic thyroid tissue sample 1 showing fetal architecture with clusters, cords and isolated epithelial oval cells, small follicles lined by cuboidal cells, and small areas of normal-sized follicles (b–h) (40x). TG: intense diffuse expression in the lumen of follicles and in cytoplasm of microfollicles and isolated thyrocytes (b). TPO: intense immunostaining in the apical membrane of micro- and normal-sized follicles; cytoplasmic TPO was observed in follicular cells and isolated thyrocytes (c). TSHR: scattered staining in the cytoplasm of thyrocytes (d). NKX2.1: strong diffuse nuclear immunostaining observed in most nuclei (e). PAX8: moderate or intense expression observed in around 80% of nuclei (f). NIS: diffuse cytoplasmic staining observed (g). Thyroxine (T4): diffuse and intense expression observed in the colloid of micro- and normal-sized follicles (h).

**Figure 2:**
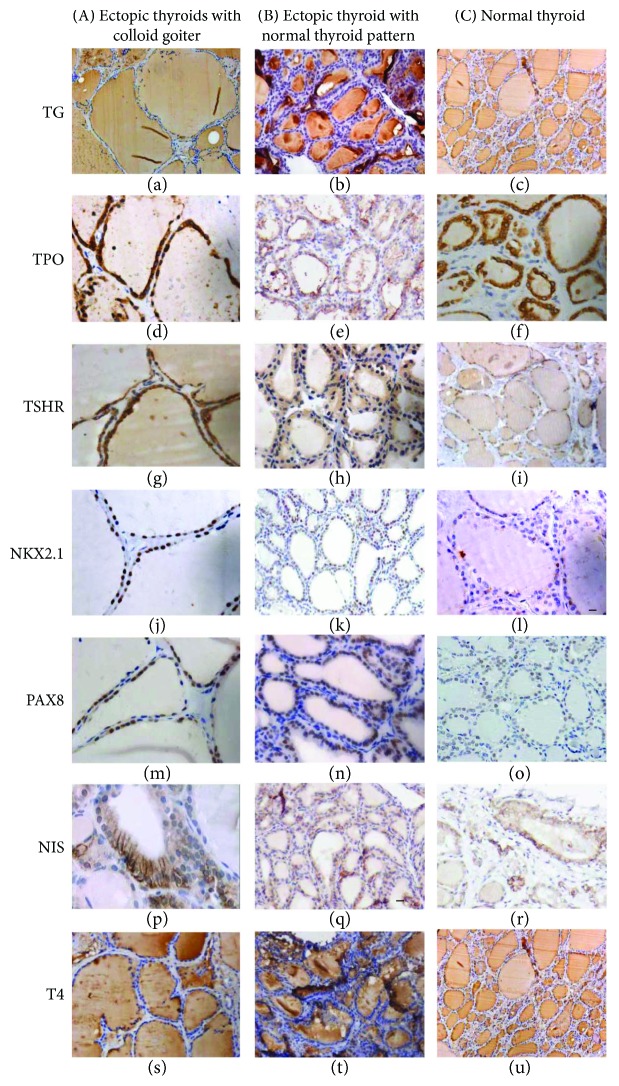
Immunohistochemistry expression of specific thyroid genes in ectopic and normal thyroid tissue. (A) Representative images of samples 2, 3, 4, and 6 showing goiter; (B) sample 5 exhibiting normal thyroid pattern; and (C) normal thyroid sample. TG: intense diffuse TG expression in the lumen of all follicles of the ectopic and normal samples; weak or negative cytoplasmic staining in cell follicles (a–c). TPO: intense immunostaining in the cytoplasm of thyrocytes and in the apical membrane of the follicles of ectopic samples examined, similar to the normal thyroid sample (d–f). TSHR: scattered staining in the cytoplasm of thyrocytes in ectopic and normal samples (g–i); magnified representative image showing basolateral membrane staining in some cells. NKX2.1: strong diffuse nuclear immunostaining was observed in most of the nuclei of the ectopic and normal thyroid tissues (j–l), except for sample 2, showing 10% positive nuclei. PAX8: moderate expression observed in around 80% of nuclei of normal and ectopic thyroid tissues (m–o) with focal areas of low nuclear expression in the ectopic samples. In sample 2, only 20% of positive nuclei were positive and scattered cytoplasmic expression was also observed. NIS: cytoplasmic staining was observed in all samples (p–r), and basolateral membrane positivity was observed in up to 10% of cells of samples 3, 4, and 5 (p, q). Normal samples had a similar pattern of 20% positivity in the basolateral membrane and cytoplasmic staining (r). Thyroxine (T4): moderate or intense diffuse expression in follicular colloid was observed, although some follicles showed low immunostaining, similar to the normal thyroid (s–u). Magnification at 40x except for the images of a, c, s, and u at 20x.

Fetal thyroid architecture was observed in sample 1 which exhibited isolated epithelial oval cells that were unpolarized or arranged in clusters and cords and primitive small follicles lined by cuboidal cells with a small lumen, some of them with colloid embedded in stroma (Figures 1 and 2). Small areas of the section with normal-sized follicles were also observed.

Histopathological examination of samples 2, 3, 4, and 6 revealed colloid goiter with highly enlarged follicles lined by flattened epithelial cells (Figure 2(A)). Focal areas (10–25%) composed of normal-sized follicles with cuboidal cells were also detected in these samples. Sample 5 showed normal thyroid tissue (Figure 2(B)).

### 3.2. Immunohistochemistry Expression

TG expression was intense and diffuse in the lumen of all follicles of the samples under investigation, even in microfollicles from tissue with fetal architecture (sample 1). Cytoplasmic TG staining was weak or negative in the normal-sized follicles and also in the macrofollicles of the ectopic samples, similarly to normal thyroid sample. However, strong cytoplasmic staining was observed in isolated thyrocytes and microfollicles from the fetal thyroid tissue (sample 1) (Figures 1(a), 1(b), and 2, a–c).

TPO staining was strong and diffuse within the cytoplasm of thyrocytes and was also observed in the apical membrane of all follicles of the ectopic samples examined, similar to normal thyroid tissue. Only cytoplasmic TPO was observed in isolated thyrocytes from fetal thyroid tissue (sample 1) (Figures 1(c) and 2, d–f).

TSHR expression was intense and scattered within the cytoplasm of thyrocytes of the ectopic samples, akin to a normal sample (Figures 1(d) and 2, g–i).

NKX2.1 diffuse immunostaining was strong in the majority of the nuclei of the ectopic thyroid samples, except for sample 2 which had approximately 10% low positive nuclei and scattered cytoplasmic expression. Low expression was observed in most of the nuclei of normal thyroid (Figures 1(e) and 2, j–l).

PAX8 moderate to intense diffuse expression was observed for all ectopic samples in approximately 70–80% of follicular cell nuclei, except for sample 2 which had a low expression in around 20% of nuclei and scattered cytoplasmic expression. Expression in normal thyroid tissue was low in most of the nuclei (Figures 1(f) and 2, m–o).

NIS was positive in the basolateral membrane in up to 10% of the cells of ectopic samples 3, 4, and 5. Cytoplasmic staining was observed in all samples. Normal thyroid sample had a similar pattern of 20% positivity in the basolateral membrane and cytoplasmic staining (Figures 1(g) and 2, p–r).

T4 expression was diffuse and intense in colloid of micro- and normal-sized follicles from the fetal thyroid sample (Figure 1(h)). In the other ectopic samples, the colloid T4 expression was moderate or intense, although some follicles showed low or negative immunostaining, similarly to normal thyroid (Figure 2, s–u).

### 3.3. Whole Exome Sequencing

After filtering the sequencing data, 3501 and 3287 variants (indel or SNVs) were identified in samples 2 and 4, respectively. The approaches to select deleterious mutations were (a) identification of common variants, (b) investigation of homozygous alterations (161 and 165, resp.) due to a possible recessive pattern of inheritance, (c) the candidate gene approach along with a list of 190 genes related to development and thyroid function (Table 1) [22–34] because no family members were available, and (d) identification of variants in the 5'UTRs of genes with binding motifs for FOXE1 (Table 2) [35, 36] and PAX8 (Table 3) [37] because these transcription factors are expressed from the early stages of thyroid embryogenesis. None of the selected variants were predicted deleterious by the specific programs. Thus, this analysis did not identify somatic mutations in the sequenced regions that could correlate with the disease.

## 4. Discussion

Ectopic thyroid has a prevalence of 1 : 100,000–300,000 individuals and 1 : 4000–8000 among patients with thyroid disease [7] and is more frequent among females (65–80%) [38] while age at diagnosis ranges from the neonatal period to adult life. In terms of thyroid function, patients can present hypothyroid, euthyroidism, and, more rarely, hyperthyroidism [7, 14, 39].

In the present study, we investigated six ectopic thyroid samples: four were lingual thyroids, one was located at the oropharynx, and another at the hyoid bone. The histology of previously reported ectopic thyroid cases have shown a normal thyroid follicular pattern [10, 40] and a predisposition for similar abnormalities to those observed in normal-positioned glands, such as follicular adenoma, colloid goiter, and others [14, 41–45]. In the present study, the histopathological examination of the ectopic thyroid tissue from one adult patient (sample 1) disclosed an immature fetal architecture pattern characterized by the presence of clusters and cords of epithelial oval unpolarized cells, primitive follicles, and small follicles embedded in stroma with small areas of normal-sized follicles [27, 46]. Thyroid tissue with fetal-like appearance was previously identified in an apparently unaffected mother of congenital hypothyroid children associated with a heterozygous *PAX8* mutation [47]. Although no mutational investigation was performed, the immunohistochemistry examination showed strong nuclear PAX8 expression in around 80% of the cells of this tissue. It remains to be investigated whether this is a functional protein. The other ectopic samples exhibited normal thyroid features (sample 5) or colloid goiter patterns (samples 2, 3, 4, and 6).

All ectopic samples expressed T4 and the specific thyroid proteins TPO, TG, TSHR, PAX8, NKX2.1, and NIS, although focal follicles, tissue areas, or thyrocytes within follicles failed to express several of these proteins (data not shown). A heterogeneous expression pattern was also observed in normal thyroid tissues and has been related to morphological features and the ability of thyrocytes and follicles to concentrate iodine [48, 49]. Sample 2 had low nuclear expression of PAX8 and NKX2.1 and scattered cytoplasmic immunostaining; however, no somatic mutations were detected by WES. Both genes have been linked to thyroid development [3] and are expressed in the thyroid anlage when migration initiates [50], and mutations have been associated with few TD cases [51]. On the other hand, in ectopia, high PAX8 expression was associated to the activation state of the gland [22], and high expression of NKX2.1 was related to abnormal embryogenesis [14]. Thus, further studies assessing the significance of the observed data are warranted.

The location of the specific thyroid proteins was similar to that observed in normal thyroid [22, 52]. The cytoplasmic TSHR staining may have been due to the use of an antibody against the extracellular domain of this protein, as previously described [53]. Likewise, the cytoplasmic NIS localization in all the samples and the membrane immunostaining in four of them was in accordance with previous reports [27, 46, 54].

Previous transcriptome analysis showed no difference in the expression of specific thyroid genes between ectopic and normal thyroid tissue when corrected for TSH levels [22]. Despite the protein expression data not being corrected for TSH levels, our results confirm the expression and the appropriate localization of these proteins in ectopia, including T4. More interestingly, they confirm that primitive follicles in sample 1 were able to synthetize T4, asserting the functional status of the gland, although it may have been unable to respond to TSH.

The clinical data of the patients suggest that the ectopic thyroids have been able to synthetize adequate amounts of thyroid hormone at least early in the patient's life. Nevertheless, the amount of secreted thyroid hormone might not be sufficient to meet the body's needs during growth, promoting hypothyroidism, given that hormone requirement is based on body weight [55]. The low hormone production may be a consequence of the small size of the ectopic glands, possibly due to the migration defect, and of a limitation in TSH-induced growth [14].

Human thyroid morphogenesis begins at 22 days of pregnancy and is largely complete when reaching its final position in front of the trachea at 7 gestational weeks (GW) (48 days of pregnancy). At this point, the thyroid is composed of undifferentiated thyrocytes and expresses strong PAX8 and weak NKX2.1 and FOXE1 [2, 50]. Subsequently, the structural and functional differentiations of the thyroid ensue, accompanied by precisely timed expression of the specific thyroid genes and can be divided into three stages. First is the precolloid stage (7 to 10 GW), with a large number of cords or clusters of undifferentiated thyrocytes expressing PAX8, FOXE1, NKX2.1, and cytoplasmic TG and TSHR, while expression of NIS and TPO is not observed. Second is the beginning of the colloid stage (10 to 11 GW) with developing small follicles with polarizing thyrocytes that express TPO in the apical membrane, TSHR in the basolateral membrane, NIS in cytoplasm, and TG in follicular lumen and culminates with T4 synthesis. The third stage is the follicular growth stage (from 12 GW forth) with the basolateral membrane expression of NIS [27, 46]. Thus, our data allow us to hypothesize that at least three different impairments in thyroid development may have led to ectopic thyroid. In patient 1, a premature arrest of migration (33–48 days of gestation) may have led to positioning of the gland at the oropharyngeal level [1] with a subsequent arrest of the structural differentiation during the early colloid stage [1, 46], resulting in a nonhomogeneous fetal thyroid tissue pattern able to synthetize T4. Second, in patient 2, the migration of the developing gland may have also stopped before reaching its final position, resulting in the thyroid positioned at the hyoid bone, along with complete structural and functional differentiation. Third, the thyroid primordium may not have been able to detach from the pharyngeal floor and migrate, promoting a lingual thyroid with a complete structural and functional differentiation as observed in patients 3, 4, 5, and 6. Thus, the arrest of this process may have occurred earlier, at 26 days of gestation, when migration begins [1].

To assess potential somatic genetic causes of TD in protein-coding regions and 5'UTRs, expanded exome analysis was performed in samples 2 and 4. Unfortunately the poor quality of the DNA from paraffin tissues from the other samples did not allow performing the genetic analysis. Despite the different exome-sequencing analysis approaches, no relevant somatic mutations were identified in the sequenced regions. A similar result was also obtained in a previous study that investigated somatic mutations in monozygotic twins discordant to TD [56]. On the other hand, WES identified novel missense mutations in the BOREALIN gene in a consanguineous family with TD [16] and DUOX2 mutations in the N-terminal domain that may play a role in thyroid development in sporadic TD cases [17], confirming the genetic heterogeneity of this pathology. Furthermore, the involvement of other genetic mechanisms may be also considered. Novel copy number variations (CNVs), not described in the healthy population, were identified in 8.75% of 80 TD patients [54, 56]. No differences in the CpG island methylation of promoter regions were observed between normal eutopic and ectopic thyroid tissues [22, 57]. Thus, the cause of TD could be associated with molecular alterations in other regions of the genoma or with polygenic or other epigenetic alterations, or it could be purely stochastic.

In conclusion, no somatic mutations in the sequenced regions were associated with ectopic thyroid. This is the first description of fetal histopathological architecture in an adult ectopic thyroid due to the arrest of structural differentiation of the developing gland along with the arrest of migration that proved able to synthesize thyroid hormone but not to respond to TSH. We showed the ability of the ectopic thyroids to express specific thyroid genes and T4, despite the migratory and histological differentiation defects, as well as the ability to synthesize the thyroid hormone for at least some period of the patient's life.

## Figures and Tables

**Table 1:** Candidate genes related to development and thyroid function (gene symbol) [22–34].

ABCA13	DIO2	FOXE3	KDR	SFRP1
ACP2	DKK3	FOXQ1	KLF4	SFRP2
ACTA1	DMD	FRS2	KPNA4	SFRS2
AKT1	DTX4	FRS2A	LAMA4	SHH
AKT3	DUOX1	FRZB	LEFTY1	SIX1
ANKRD36B	DUOX2	FXR1	LHX3	SLC26A4
ARHGEF6	DUOXA1	FZD1	LHX4	SLC5A5
ASPM	DUOXA2	FZD3	LMO3	SMAD3
ATP2A1	DUSP6	FZD4	LRP8	SMAD5
BCL2L1	EDN1	GATA5	LYZ	SMAD9
BCL2L12	EDN3	GJA1	MKI67	SMOC2
BGN	EEF1A2	GLIS3	MKRN1	SNX1
BMP4	EFNB2	GNG5	MS4A6A	SOX17
C9orf70	EGFR	GPNMB	MTHFD2	SOX9
CCND1	EGR1	GSTT1	MUC1	SPRED1
CDC42EP4	EMP3	HADHA	MYBPC1	TAL1
CDH16	ENO3	HAND2	MYL2	TAZ
CDH2	EVC2	HES1	NAV1	TBX1
CEBPB	EYA1	HESX1	NKX2.1	TCAP
CECR1	FAU	HHEX	NKX2.5	TCF4
CFC1B	FBLN1	HLA-DQA1	NLK	TEF
CGA	FGF10	HLA-DQB1	PABPC1	TG
CHGA	FGF12	HOXA2	PAX2	TGFB2
CHORDC1	FGF2	HOXA3	PAX8	THRA
CKM	FGF3	HOXA5	PBX4	THRB
CLDN5	FGF8	HOXB3	PCSK2	THRSP
CNTN6	FGFR1	HOXD3	PITX2	TNFAIP2
COL1A1	FGFR2	HSPA1B	PKNOX1	TNFRSF21
COL3A1	FGL2	IGHG4	PLCXD1	TNNC2
CPEB4	FLJ11127	IGJ	PLEKHA3	TPO
CREB1	FLJ32115	IGSF1	PLXND1	TRA
CTGF	FMR1	INHBB	POLD4	TRH
CTNNAL1	FN1	INSL3	PRKCE	TSHb
CXCL12	FOS	ISL1	PROP1	TSHR
CXCR4	FOSB	ISL2	RARRES1	TWSG1
CYBB	FOXA1	IYD	RASD1	TXNIP
CYBRD1	FOXA2	JAG1	RNASE6	TYROBP
DIO1	FOXE1	JAG3	ROBO4	VEGFA

**Table 2:** Target genes of the transcription factor FOXE1 (gene symbol) [35, 36].

ADAMTS9	CRIP2	ENGASE	IL23A	RT1-DA
AHCY	CTGF	ERO1LB	KRT20	S100A4
AMIGO3	DDIT3	ETV5	MANF	SDF2L1
ANKRD37	DERL3	FGF18	MFSD2	SEC23B
ATMIN	DNAJB11	FOLR1	NR4A2	SEL1L
BCAM	DNAJB9	GGCT	NUPR1	SLIT1
BET1	DNAJC3	GMPPB	PDIA4	TM4SF1
CASP4	DUOX2	HSP90B1	PRIMA1	TMEM140
CDH1	DUSP5	HSPA5	PRSS8	TMEM66
COQ10B	DYNLRB2	HYOU1	RIL	ZFAND2A
CRELD2	ELOVL2	IGF2BP2	RIOK3	

**Table 3:** Target genes of the transcription factor PAX8 (gene symbol) [37].

ACOT2	CFD	GCSH	LACTB	RASSF2
ACY1	CITED2	GJA4	LRP8	RSAD2
ADAMTS9	CRYAB	GSTP1	LRRC58	RUNX2
ALCAM	CTGF	HACD4	NFKB1	SLC26A7
ANKRD9	CXCL1	HSD17B1	NR3C2	SMIM22
ARHGAP22	DGAT2	IGFBP5	NRIP3	SPARC
BHLHE40	EGR1	IGFBP7	NUP107	STS
BRAF	EIF4E	IRGQ	OPRK1	TAZ
CAMK1G	ENPP1	JUN	OSTALPHA	TEKT4
CAMKK2	F10	KCNJ15	PBLD	TG
CD47	FAM13A	KCNJ16	POMT1	TMEM140
CDA	FGFR2	KCNK1	PRR5L	TRIB1
CDH16	FOXE1	KRT14	RAB17	WBP2
CDH16	GALK2	KRT7	RASL10A	WNT4
